# Traditional Healing and Law in Contemporary Senegal: Legitimacies, Normativities and Practices

**DOI:** 10.1177/09646639221122434

**Published:** 2022-09-13

**Authors:** Emilie Cloatre, Tidiane Ndoye, Dioumel Badji, Adams Diedhiou

**Affiliations:** 2240 University of Kent, UK; Université Check Anta Diop, Senegal; Cabinet SISTEM, Senegal; 89230 Université Check Anta Diop, Senegal

## Abstract

In this paper, we chart the context in which contemporary legal debates around traditional healing in Senegal unfold, pointing in particular to the type of power–knowledge relations that are at stake in both the current legal status–quo, and legal changes proposed in 2017. We interrogate the struggles over legitimacy and recognition that are at play in these processes, and the ways in which different actors relate to both formal legal rules, and more fluid forms of legalities, in which imaginaries of the law, and negotiations with the law, translate into everyday practices. We underline how legal and scientific discourses are mobilised to draw the opportunities and boundaries offered to different healing agents, and to organise their respective authority. Traditional healers overlap with modern health practices, while retaining their own ontologies and claims to legitimacy while representatives of the biomedical professions insist that they should have some oversight over the regulation of all healers. As negotiations continue over the possibility for the state to regulate traditional healing, everyday legal choreographies define the relative roles, possibilities and precarity of different healing agents.

## Introduction

In November 2017, a legislative proposal on traditional medicine was brought to the Senegalese Parliament. Soon after, the Ordre des Médecins (General Medical Council) and other health professionals expressed their opposition in the strongest terms. The draft legislation became the focus of a legal, and epistemological, controversy that is still ongoing: the text has not yet been adopted, and it is not clear whether it will. But it continues to animate conversations among doctors and traditional healers alike, fuelling tensions that have been ongoing for several decades, and hinge on the questions of whether traditional healing should be regulated by the state, and if so, how healing techniques and healers deemed legitimate can be separated from those considered problematic. Maybe more importantly, this debate brings to light tensions around the tacit arrangements currently in place, in the absence of formal regulation.

The 2017 debate reflects the uncertainty that characterises the interface between law and traditional healing around the world. If the WHO has increasingly embraced the idea that traditional healing should be regulated by states, such embedding of traditional healing in the mechanisms and logics of legal regulation raises numerous theoretical and practical questions (Tilley, 2021). At stake are fundamental issues about the interface between science and other forms of knowing and healing, and about the role of different groups and institutions in overseeing therapeutic care. In Senegal as elsewhere, these questions unfold in a postcolonial context that has affected both legal systems, and medical institutions (Snyder, 1981; Tousignant, 2012, 2013). Debates also rest on particular configurations, including the ambivalence of the current legal status of traditional healers, and local institutional dynamics, notably with regards to the role and political standing of the Ordre des Médecins. If the Ordre is a privileged interlocutor of the state in the regulation of medicine, and potentially other public health matters, its primary role is that of a professional association, tasked with maintaining ethical and professional standards in medical practice, including through disciplinary action. The central role currently played by the Ordre in relation to the regulation of traditional healing, and contestations around its future, illustrate the interface between institutional and epistemic legitimacy in the regulation of healing, and the ambivalent position of traditional healing vis-à-vis biomedicine . Currently, the Ordre des Médecins’s de facto role in regulating the practices of healers who rely on a different type of ontology, partly born out from a history of legal uncertainty, raises broader questions about the institutional relationship between law and science. Biomedical knowledge and its institutional authority are mobilised in our case study to fill some of the gaps and uncertainties fostered by the absence, to date, of legislation on traditional healing, leaving it instead subject to laws written for, and about, biomedicine. Recent efforts by the state to intervene more directly, erecting traditional healing as its own regulatory matter, expose the difficulties of moving away from long-standing epistemic and institutional configurations. But until these efforts materialise, legal influences over traditional healing take multiple shapes. Law is translated, interpreted, stretched to territories where it may not technically belong, and coexists with a set of other norms and expectations from which it can be difficult to disentangle.

In this paper, we chart the context in which contemporary legal debates around traditional healing in Senegal unfold, pointing in particular to the type of power-knowledge relations that are at stake in both the current legal status-quo, and proposed changes. We interrogate the struggles over legitimacy and recognition that are at play. In particular, we explore the ways in which different actors relate to legalities, both as formal legal processes, and as more abstract notions of ‘quasi-law’, in which imaginaries of the law, and negotiations with the law, translate into everyday practices. In these processes, legal and scientific discourses are mobilised to draw the opportunities and boundaries offered to different kinds of healing practices, and to organise their respective authority. Traditional healers overlap with biomedical practices, while retaining their own ontologies and claims to legitimacy. The Ordre des Médecins and (to a lesser degree) representatives of other medical professions, see the legitimacy of healing as necessarily conditional on their own intervention as quasi-legal actors. As negotiations continue over the possibility for the state to regulate traditional healing, everyday legal choreographies define the relative roles, possibilities and precarity of different healing agents.

## Approaching Traditional Healing and its Legal Ordering

This paper builds upon three distinct bodies of scholarship, to interrogate critically the interface of law and traditional healing in contemporary Senegal. First, we engage the extensive work in anthropology and Science and Technology Studies (STS) on traditional healing, particularly in its interactions with the state. Scholars have long highlighted that the terms ‘traditional healing’ cover myriad forms of knowledge, techniques, relationships, that far from being fixed in time are constantly evolving (Pordié, 2012). Even within a particular location, as is the case in Senegal, practices coexist, and patients borrow from a range of resources to seek therapeutic care (Hampshire and Owusu, 2013). In each context, the relationship of traditional healers with the state also holds its own particularities, rooted in both history and socio-cultural configurations (Wahlberg, 2006; Langwick, 2011). This has contributed to the difficulty of regulating the field of traditional healing: its object is in and of itself ‘slippery’ and multiple (Cloatre, 2019). Traditional healing also disrupts the mechanisms of evidence on which medical regulations have been built. The methods used to determine the efficacy and safety of materials and techniques of biomedicine, and organise its regulation, can be of limited use for practices that rest on different ontologies (Janes, 1999; Adams, 2001, 2002). For example, the spiritual dimension of many healing practices challenges the idea that testing the validity of healing techniques can be done by isolating body from mind, or separating the healing interaction itself from the effects of a particular remedy. As such, clinical trials, considered as the gold standard for biomedical evidence because of their ability to test blindly the physical effects of particular interventions, cannot easily be adjusted to traditions where spiritual and bodily wellbeing are seen as interlocked, and the relationship of healer and patients as central to the efficacy of the treatment given (Adams, 2002). Similarly, systems of training and qualifications, that often constitute a key aspect of the professional regulation of healthcare, may not be adapted to healing systems in which family lineage, religious callings, or informal apprenticeships are seen as more relevant signifiers of legitimacy. The regulation of traditional healing therefore requires particular forms of legal imagination, and raises difficult questions about how to adjudicate the legitimacy of non-scientific (or not exclusively scientific) knowledge, and about who should be entitled to do so.

As well as being a complex technical matter, the regulation of traditional healing has wide-ranging sociocultural ramifications. Formal regulation, here as elsewhere, is also an act of recognition, seen by defenders of traditional healing and its detractors as raising questions of belonging, and how practices and agents are inscribed by states in collective past, present and future. The positions states and legislators adopt towards healing therefore echo broader debates about identity at the crossroad of tradition and modernity, and tensions around the very definition of each of these terms (Sarr, 2016). But like in other fields, the regulation of healing is inevitably performative: in creating the conditions of legal practice, states also nudge healers to adjust or adapt, and participate in creating new lines of inclusion and exclusion, that go on to be negotiated through everyday practice. These dilemmas echo broader questions about the interface between law and science, and the workings of law in everyday society. Notably, legal debates around traditional healing provide some rich insights into how different kinds of expertise shape legal possibilities, while also being the product of regulatory influences.

For this, we build onto the work of scholars working at the crossroad of law and STS (Cole and Bertenthal, 2017; Cloatre and Pickersgill, 2020; Turner and Wiber, 2022). This includes explorations of the layered processes through which policy and regulatory knowledge is constituted, while other forms of expertise become seen as less relevant to public decision-making (Jasanoff, 1990, 1995). The institutional positioning of individuals and disciplines, the possibility of translating expert perspectives into relevant policy registers, or pre-existing sociocultural constructions of what rational knowledge looks like, contribute to such triage, which once established can become difficult to unsettle (Jasanoff, 2006; Anwar and De Goede, 2021). The type of knowledge on which law depends is also shaped by multiple influences, rather than fixed and objectively definable. Institutional and social histories, as well as evolving sociocultural and legal relationships, participate in delimiting what becomes considered as valid knowledge by regulatory institutions. In the context of medicine, we can argue that institutional dynamics create an inherent challenge for non-biomedical forms of knowledge, and this is visible in the context of Senegal. The entanglement of legal and scientific institutions and logics, in the institutional make-up of medicine, can dwarf other ontological possibilities (Adams, 2002; Cloatre 1 2019). States may see biomedical experts as the primary holders of knowledge over bodies and healing, best placed to intervene in the regulation of medicine. This comes to determine how products, procedures, or professionals are organised, ordered, or triaged (Gaudillière, 2013). But in biomedicine and its regulation as elsewhere, the making of knowledge is entangled in questions of industrial production, global markets, or laboratory processes that all come to define the finer translation of ideas into practice (Tilley, 2021). This entanglement of state decision-making, regulatory choices, and scientific knowledge complicates the negotiation of traditional medicine as viewed by the state. Traditional healing comes to represent a challenge to the scientific and legal settlement, by proposing that other ways of knowing and healing may also have a role to play in the delivery of healthcare, and therefore need to be confronted and made to fit in legal mechanisms and expectations of evidence, verification and knowledge-ordering (Wahlberg, 2014).

Finally, our study explores a moment of legal controversy, against a background of regulatory uncertainty. In studying both aspects, we rely on broader socio-legal scholarship, at the crossroad of legal consciousness and legal pluralism (Silbey 2005; Cowan 2004; Merry 1988), in addition to work on law and STS. In the processes we studied, the law is performative, but also always in translation: it is adjusted and rewritten through practice, engaged with lay-users in ways that rewrite its own limits and possibilities (Silbey, 2005). If law creates easier paths for some actors than others, it always leaves open a possibility of negotiation (Cloatre and Enright, 2017). This negotiation can take several forms, from boundary-stretching to the mimicking of legal compliance, even when such compliance is not formally expected. The negotiation of legality and illegality is also a process of determining future opportunities, and of reclaiming visibility: in this way, the act of coexisting with the law also involves tacit political, and at times ontological claims (Hertogh and Kurkchiyan, 2016). Here, as they seek to define their place in the visible landscape of healthcare, traditional healers adopt legal behaviours that mimick those imposed on doctors, while being expected to remain on the edge of the law, rather than entering its spaces. The law is loaded with unexpected effects, including through the performative value of compliance (where being seen to comply with the law, even when it is not required, enhances legitimacy), and the processes of translation that take place where legal texts navigate through lay networks of practice (Hertogh, 2004; Cloatre and Enright, 2017). In these processes, legal norms bear complex relationships with state law, deriving in part from it and in part from other forms of ordering. The messy circulation of legal rules and their layered coexistence with other normative systems, form lived expressions of legality (Young, 2014). While focusing on these matters in the very particular context of healing, this paper therefore also illustrates broader questions about the distribution of legality, and forms of living law.

This research in Senegal is part of a larger project, that looks at the dilemmas that arise in attempts to regulate alternative and traditional medicine, through selected case studies in Europe and Africa. Rather than adopting a particular normative stance, about specific healing techniques or paradigms, it seeks to provide a critical analysis of the challenges that arise when states set out to organise practices that rest of paradigms other than those of science and biomedicine. To this effect, our research in Senegal is based on qualitative methods, including interviews and documentary and legal analysis. It was carried out between 2018 and 2021. Ethical approval was obtained from the University of Kent (in 2016) and from the Comité d’Ethique sur la Recherche en Santé du Sénégal (in 2018). We carried out twenty-two interviews with actors involved in or affected by ongoing debates on the regulation of traditional healing, in three successive phases, with initial interviews and mapping out carried out by EC, in French and further interviews by DB and AD in French and Wolof. Our participants included key policy actors involved in the debates on the regulation of traditional healing (including from the Ministry of Health, the Ordre des Médecins and Ordre des Pharmaciens) or whose duties involve the monitoring of health practices or authorisation of health products. We also interviewed representatives of healers associations, of the main institutionalised centres of traditional medicine (Fatick and Keur Massar) and a small sample of individual healers. Finally, we interviewed actors with long term knowledge or experience of the field, including in research centres and NGOs. Interviewees were initially identified from documentary research, including policy documents and media reports, and later through snowballing, contacted initially by phone, or in a few cases by email. Most of the actors we aimed to contact were willing to speak, with a few exceptions where we could either not locate particular individuals, or not secure an interview. In order to preserve anonymity, we have had to maintain references to interviewees we quote fairly general, particularly where their function or affiliation would make them easily identifiable. Our interview data was supplemented by documentary analysis of legal and policy documents relevant to traditional medicine, with a particular focus on the 1990s onwards. These are contextualised by engaging with relevant historical and sociological literature on the practice of traditional healing in Senegal.

## Traditional Healing in Senegal

Like in other states in West Africa, traditional medicine has a long history in Senegal, and healers continue to play a significant part in everyday access to healthcare (Fassin and Fassin, 1988; Simon, 2004). Initiatives by the state since the late 1990s, including the ongoing attempt to regulate traditional healing that we return to, have sought to cement a distinctive identity for contemporary traditional healing. Efforts have been made to integrate traditional healing into other forms of healthcare through small community projects, and from the turn of the millennium, a series of government initiatives aimed more explicitly to rethink its potential value. In particular, such effort focused on establishing scientific credentials for traditional healing, disentangling ‘unproven beliefs’ from materials and practices that could stand up to the tests of modern science.^1^ Research into traditional healing has been encouraged, symbolised for example by the inclusion of a chapter on traditional healing in the 2009 law creating the *Code d’Ethique pour la Recherche en Santé* or the activities of the Traditional Medicine Unit of the Ministry of Health.

Within such initiatives, the potential future of traditional healing, and its inherent value, are at the same time practical and symbolic. At one level, the aim is to improve practice: ‘*initially it aimed to help healers, to bring scientific support to improve what they give to people, because whatever we do, they are part of our culture and people will go to see them*’ (interview with a public health official). But the symbolic significance, folding into a broader emphasis on the value of local knowledge and resources to national identity, is also notable. As one interviewee from the Ministry of Health deplored: ‘*here we have Chinese or Indian traditional medicine, anyone can come, and we take whatever they bring. We accept all this, but what is done here by the Senegalese is neglected*’. Another policy actor framed this idea of revaluing in these terms: ‘*We need everyone to know that the future of medicine is traditional medicine, because nowadays it is all fine to import medicines from elsewhere, neatly packaged in factories, but we also need to recognise that Africa has gifts. We are Africans, and there is a reality here. So products that are here, and that collectively we agree can heal, we need to work on improving them and conditioning them appropriately*.’ In these discourses, traditional healing is one component of a particular kind of socio-technical imaginary (Flear and Ashcroft, 2021), proposing a future for healthcare that is more determinedly Senegalese, yet fits into a concept of modernity seen as universal, and as scientific.

Against this background, the everyday of traditional healing in Senegal offers a diverse picture: rather than projecting a particular ambition or direction, it adopts different forms, mapping partly onto the significant religious, ethnic and geographical diversity of the country, while also being affected and adjusted through the practice of each individual healer (Diedhiou, 2021). While some healers embrace the government’s idea that the scientific potential of traditional healing needs to be harnessed, others understand their practice as operating in a very different sphere from that of science or biomedicine. Spiritual practices, in particular, may not be seen as relevant to governmental ambitions to foster the scientific potential of local plants or techniques, yet underpin much everyday practice. The public profile of healers and their links to local community are similarly varied: some practice only locally, within their own community, while others use the media to gain national or international visibility or to set up practice across borders (Faye, 2011). For the latter, the financial benefits derived from such activities may be at the cost, however, of their legitimacy vis-à-vis other healers, who consider such proactive advertising and media visibility as suspicious and unethical. Training and personal trajectories are hugely diverse, with some healers having inherited their skills and knowledge from family lineage, while others were trained later in life, formally or informally. Myriad practices of healing coexist, straddling the boundaries between imaginaries of tradition and modernity, borrowing tools, language, practices, and techniques from old and new, customs and science, some engaging with doctors while others see their roles and ethos as too different to overlap. Traditional birth attendants, herbalists, spiritualists, new age healers or traditionalists, offer services ranging from the benign to the spectacular, and from specialist to generalist.

Healers are also organised in groups and associations, both formally and informally, where experiences can be shared, mutual interests protected, and a sense of shared ethics developed. Associations also act as markers of legitimacy, particularly in fostering relationships with the government: associations provide an anchor point when agents of the state seek an interlocutor, for instance to discuss the form that regulation could take. Alongside associations, two institutions represent a different type of institutionalisation of traditional healing in Senegal. The Hôpital Traditionnel de Keur Masar, created by French researcher Yvette Paré in 1980, positions itself as a site of specialist traditional care. Healers work alongside each other, their practice rooted in tradition yet shaped by shared rules and procedures. Medicinal plants are conditioned, packaged and labelled on site, and healers prescribe them to individual patients after consultation. Supported at the time of its creation by academics and state actors, initially as a site to treat lesprosis, the hospital represents a particular form of healthcare delivery modelled on biomedical hospitals, while using local plants and hybrid methods (Fassin and Fassin, 1988). The Centre Experimental de Fatick is a different kind of institution, with a different set of claims, at the crossroad of tradition and what others have described as New Age healing (Simon, 2003). Its vision is more explicitly turned towards research and development, and its outputs have included highly controversial claims (Simon, 2004). It is also more openly political, seeking a global recognition of African knowledge as part of a broader identity project built around a transnational network of activities. The Centre also has considerable influence at the national level, including in lobbying for the legal recognition of traditional healers. Even though, as Fassin and Fassin (1988) highlighted already in the late 1980s, these two institutions illustrate the contradictions in legitimacy that cut across traditional healing: if they stand as obvious interlocutors of the government, and are involved in most conversations about the future of healing, they are also very particular actors of the everyday of traditional healing, bearing limited resemblance to the much broader social spectrum of healing that they are seen by some as representing.

Overall, legitimacies are layered and relational among traditional healers: with no current top-down state regulation, different codes, customs and normative expectations coexist to organize, in ways that may appear loose and informal, yet are inscribed in the current network of practices. Yet, and as we turn to, with no formal state recognition of any of the standards and principles that organize current practices, healers are both precarious, and unable to filter out those that they see as posing a danger to professional standards, and to patients.

## Current Legal Context: Healing, Biomedicine and Otherness

Despite its centrality to everyday healthcare, traditional healing in Senegal has not to date been formally regulated by the state. Although Senegal is by no means unique in not having specific legislation on traditional healing, its position is increasingly unusual in the region: in West Africa as elsewhere, traditional healing has increasingly become seen as a regulatory matter, echoing a framing favoured by the WHO particularly since the early 2000s (Cloatre and Ashworth, 2022). Senegal meanwhile has been taking more modest steps towards legal change. In 2017 a long-awaiting legislative proposal was adopted by the Ministerial cabinet, laying the foundations of a proposed new organisation for traditional healing. But, following acrimonious criticism, this proposal has since failed to be translated into the new law that Ministers had hoped to see.

In the absence of explicit regulation on traditional healing, its boundaries are framed indirectly. Some of its borders are set as a result of laws on illegal medical practice, that participate in determining what spheres of activity are under the exclusive control of biomedical professionals. Others are negotiated through professional practices and ethics. Following these legal and other normative threads, however, suggests cross-fertilisation and negotiations between legalities and layered legitimacies, that throw into question the boundaries of what should be considered as ‘legal effect’ and what belongs to other social orderings, and the relevance of such distinctions. More pertinent, and cutting across these different orderings, is a constant negotiation of the social positioning of traditional healing, alongside, or against, biomedicine. The attempt to formally regulate healing called for a redefinition of this positioning, fuelling epistemological and jurisdictional conflicts between state, healers and doctors. Traditional healing is both seen as a cultural resource whose value (social, economic and health-related) could be harnessed by a different form of state engagement; and as a field in need of ‘tidying up’, where the state needs to contribute to disentangling reliable healers from less trustworthy ones, and efficacious remedies from others.

### Informal regulation at the border of medicine

Like other fields that are not formally regulated by the state, traditional healing is framed by a number of, implicit or explicit, shared norms and principles that a majority of healers seem to agree upon, even when their practices differ otherwise (Oyebola, 1981; Fassin and Fassin, 1988). While those norms have emerged and been stabilised over time, they are also subject to the type of ongoing patterns of settlement and challenges that formal law experiences. While an in-depth exploration of the ethical and professional principles that order traditional healing as a field of social practice is beyond the scope of our study, a few elements were repeatedly put forward in our interviews, that we flag up – while acknowledging their incompleteness – in order to contextualise our discussions of formal legal relations. For example, healers we met considered it as their duty to protect patients from harm. To that effect, they insisted that healers should always be clear about the limits of their own knowledge. Vice versa, healers who claimed to be able to cure any illness were portrayed as likely to be dishonest. Similarly, those we met shared a general wariness towards healers who make significant profit from their healing practice, since healing should not be primarily for financial benefit: as one interviewee put it ‘*real healers are not rich!*’. Such principles can overlap with tactics employed to avoid being caught in the net of illegal medical practice, that we return to below: for instance, healers we met were critical of those who extensively advertise their services both as a matter of ethics, and because they were unsure about the legality of advertising. The need to be modest in one’s claims was both presented as a sign of professionalism, and as a condition to remain tolerated despite legal precarity. Similarly, a focus on avoiding harm to patients was seen as both a good in itself, and essential to protect oneself from the judicial system. Shared norms and ethics, serve as one set of markers of legitimacy, towards patients, and within a community of healers. They coexist with other such markers: for instance, family lineage, local reputation, training, are seen by some healers as essential in determining who is a ‘good’ healer from those that they might accuse of quackery.

The set of customary and professional norms that frames traditional healing does not operate in a vacuum, but also echoes some of the regulatory norms that apply to other healthcare professionals. Healers can be likened to a semi-autonomous social field, in the phrasing of Sally Falk Moore: legitimacy and legality, customs and legal norms, coexist through myriad nodes, shaping each other and shaping traditional practice in ways that are not always possible to disentangle. The informal norms that apply to healers are also shaped by legal regulations that indirectly determine the possibilities open to their practice, and the fluid interpretation of these norms. Centrally, these stem from the legal imperative for healers of not impeding on the sphere of medical practice (however fragile its definition may be), and how to exist at its border.

In 1966, Senegal adopted a law organising the legal practice of (bio)medicine. The law sets out the conditions required to practice legally as a doctor, and conversely sets out details of the criminal offence of ‘illegal medical practice’. The latter applies to ‘*anyone who takes part in the diagnosis or treatment of illnesses or surgical conditions, established or suspected*’. This echoes the language used in a 1922 French colonial law, and indeed that used to this day in the French law on illegal medical practice. The potential scope of this phrasing is broad, and creates a degree of uncertainty over what constitutes ‘diagnosis’ or ‘treatment’, and indeed over who should be best placed to define the legal scope of these terms (Kuitche Kamgoui, 2004). In theory at least, much of the activities of traditional healers could be considered as either diagnosis or treatment: in France, by comparison, the law on illegal medical practice has been applied to non-medically qualified healers practicing alternative or traditional therapies (eg. such as acupuncture) (Candelise, 2010; Parent, 2015; Cloatre, 2018). The Senegalese Ministry of Health, in its introduction to the 2017 legislative proposal that we return to, begins by stating that the 1966 law ‘*does not allow the legal practice of traditional healing in Senegal*’.

In practice however, the legality of traditional healing appears more nuanced: a degree of tolerance is granted to traditional healing, and the laws on illegal medical practice are not regularly used against traditional healers. Straddling the boundary between health and cultural practice, and usually affirming its difference from biomedicine, much of traditional healing is seen as not crossing into illegal practice. It operates within a zone of legal tolerance, only occasionally meetings its limits. In what follows we explore how the boundary between tolerance and intervention is constructed in the day-to-day, focusing on its ordinary dimensions rather than the more extraordinary – and rarer – cases of prosecution of healers because of the harm they might have caused.

### Tolerance and Othering

A particular feature of the legal order is the central role of the Ordre des Médecins in determining the everyday boundary of tolerance: if prosecutors are able to initiate any action they see appropriate, denunciations mostly originate from the Ordre (eg. Senenews, 2022). Drawing the attention of the judicial system to any activities that encroach onto the sphere of their exclusive legal practice is seen as a part of their duty to ensure the safe boundaries of medicine, while protecting its sphere of practice and reputation. Legal boundaries are therefore heavily shaped by the judgments and definitions of a biomedical profession, elevated as a quasi-regulatory order not only over biomedicine, but also beyond it. Although the Ordre is most vocal in its framing of boundaries, and most active in monitoring that these are not crossed, their reading is shared by other health policy-makers whose focus is primarily on biomedicine.

Following the translations of the law into practice illustrates the inherent ambivalence of the relationship between law, medicine and traditional healing, fomented by the role of doctors in adjudicating the borders of legitimate healing. Traditional healing is caught between a demand that it asserts its difference in order to be tolerated, and an expectation to comply with the standards and norms that doctors are accustomed to. It operates as a form of regulatory boundary object, maintaining a fragile position between legality and illegality as different actors project their own expectations and readings onto it. We illustrate this through two main zones of friction between the law on illegal medical practice and the activities of traditional healers.

#### Tradition as Difference

For the Ordre des Médecins, and indeed other health policy-makers, the legitimacy (and legality) of traditional healing is rooted in its socio-cultural significance. But this is conditional on healers undertaking only ‘genuine’ traditional activities, without encroaching onto the spaces of biomedicine. Summarising the difference between traditional healing they deem acceptable or not, one policy-maker explained:

“If you are at home, you receive some visits, noone will come bother you. But if you show off, on the market or whatever, or just opposite the dispensary, or if you write prescriptions, go on the radio, then we will ask you questions. But if you practice traditionally, noone is going to go to Thionk Essyl [town in the Casamance region] to ask ‘where did you learn this from?’”

The emphasis is both on discretion, and on a clear distinction from the spaces and activities associated with biomedicine (with references to dispensaries and prescriptions). This distinction is key in determining the boundary between tolerable activities, and those perceived as illegal: where healers are seen as getting too close to what belongs to medicine, they are less likely to be tolerated by the Ordre and those who share their views. From this has emerged the most visible source of friction between the Ordre and healers: some contemporary healers, keen to use all the resources available to diagnose their patients, occasionally invite them to undertake biomedical tests – X-rays, blood tests, ultrasounds etc. The results of the tests contribute to determining which traditional remedies will be offered. For the Ordre des Médecins, this represents the type of encroachment onto biomedicine that signals illegal practice, and justifies a denunciation. Healers are aware of this risk, yet often sceptical as to its fairness and rational basis. Telling us about another healer who ‘*got in trouble with the Ordre des médecins*’, one interviewee therefore explained that *‘when a patient came to see him, and he didn’t know what they had, he would write a paper to tell the hospital that he was sending them over. Doctors used this to say that he was not allowed to write the paper because he is not a doctor […] But this was just ‘i’m sending this patient to see if he has an ulcer or hepatitis B…’ and they tell him he shouldn’t*?’

That tools of diagnosis are particularly contentious suggests a specific stake of boundary-making. Usually, such boundary-setting by biomedical institutions hinges on notions of risk or harm, for example when products are given as treatment by unqualified practitioners. This is less explicit in this case, where the concern is arguably over an act of ‘knowing’ rather than ‘doing’. The concern at stake is one of blurring the boundaries between what should (at least for the Ordre des Médecins) remain clearly distinct systems, with different ways of both diagnosing bodies and ultimately treating them. Letting traditional healers use diagnostic tools suggests an intrusion onto the epistemological resources of biomedicine, and the expression of a different kind of knowledge over the body, that makes them potentially more problematic in the eyes of the Ordre, and those who share their views.

Despite their puzzlement, healers have sought to adapt to this boundary: they are cautious in suggesting rather than prescribing medical tests, and may for example ask patients to write down details of the test to be requested, rather than write it themselves (so that it is harder to claim that the test was ‘prescribed’ by the healer). Practitioners operate along very fragile lines of legality and legitimacy, and the law itself becomes translated through a tacit form of everyday negotiation.

As the president of one association of healers explained : *‘what i try to underline with healers, in general, is that their problem tends to come from writing diagnosis, or recommendations for tests. Ah, that’s all the Ordre des Médecins are waiting for! As soon as you write that down, they take you to court ! Or if you write ‘ultrasound’ and you put your stamp, that’s it ! they sue you for illegal medical practice, misused professional identity, all of it!’*

The practices at stake are a stark reminder of the difficulty of defining ‘traditional’ healing: healing practices fluctuate over time, rarely confined to an imagined past, and healers borrow techniques and tools from a range of knowledge-systems, reinventing their practice to respond to new demands and opportunities. Interactions between traditional and biomedicine fluctuate, creating new frictions and possibilities (Duchesne, 2021). In Senegal, these processes become complicated by the pre-existence of legal tools that effectively enable biomedical institutions to monitor the boundaries of their professional monopoly, delimiting what they consider as being unacceptable intrusions and, in that process, framing the possibilities that are open to other traditions. These legal tools, as we saw, are directly inherited from the colonial period: but it is not just the text of the law itself that has marked this governance system. The entanglement of biomedical and legal institutions also forms part of this regulatory inheritance (Badji, Devaux and Gueye, 2013). Through the scope it leaves for interpretation of what may constitute an illegal medical act, the law opens-up epistemological tensions of which the Ordre des Médecins is the most central arbiter, and the de facto key agent of legal boundary-setting. While formally established primarily to govern the medical profession, it is able to extend its power to a tight monitoring of those who are, precisely, not doctors (Freidson, 1970).

#### Mimicking Legality

The frictions caused when healers reach out to the diagnostic tools of biomedicine suggest that their legality is conditional on their distance from biomedicine: they are tolerated because they are Other. Yet, in other respects, their practice within a zone of ‘healthcare’ means that they are also expected to submit to the rules that are imposed on biomedicine, even when these do not formally apply to healing. Navigating between Otherness and Sameness, healing is caught in a fragile set of norms, shaped by a tacit consensus between healers, policy-makers and doctors, yet lacking the clarity that formal regulation could provide. Illustrating this, a second set of friction between (some) healers and the Ordre des Médecins, but also among healers themselves, is the question of advertising. Several texts prohibit explicitly advertising in the context of medical practice in Senegal: the Code de Déontologie Médicale (Art. 10) prohibits doctors from advertising their services, and the advertising of medical products is also prohibited (Loi 65-33 du 19 Mai 1965). Yet it is not clear that the scope of either legal document should extend to traditional healers: the scope of the Code de la Déontologie is limited to medical doctors, and their conditions of practice. And the 1965 law defines ‘medicines’ for its purpose as those that are sold in pharmacies (which is not the case for most traditional medicines). The Code de la Presse also reiterates, since 1997, the prohibition of advertising of medicines (though limiting it to ‘prescription medicines’)^2^. It contains an article (art. 106) that may seem more relevant to the type of practices that traditional healers are considered as undertaking, and bans ‘misleading advertising’. Yet, when advertising by healers is decried by either policy actors, doctors or traditional healers themselves, references are not to this latter provision, but inferred from the prohibitions that are imposed on biomedical professionals. For example, in 2019 the Ordre des Médecins wrote to the CNRA to ask for their support in preventing healers from advertising their services and products, reminding the CNRA that this is in breach of the Code de Déontologie, and that it is ‘in this context’ that the Ordre has sought proceedings for illegal medical practice against some healers^3^. In our interviews, references tended to be made to a more general principle of prohibition of advertising in medicine that healers should, like doctors, be expected to follow, as well as to the risk that such advertising might create. For example, one representative from the Ministry of Health explained that they were ‘*in agreement with people who protested, recently, against what we see on TV, on the radio: advertising, people who say they can heal anything. There are shows dedicated to them, but are any of the information they give verified? I think we really need to control such advertising because there is no scientific backing to any of it*.’

If a minority of healers are heavily reliant on advertising (arguing, for example, that this is the only way for them to be known) others look down upon the practice, echoing policy actors in their judgment, though not necessarily in their rationales. Their concerns are primarily about legitimacy: healers who need to advertise their services are not considered ‘*sérieux*’/[professional]. But not all healers in our interviews attributed this issue to legal prohibition. For example, one healer explained that ‘*In France now they advertise Doliprane and stuff, but here, in Senegal, it is prohibited, except for traditional healing because that’s not regulated, so some radio stations agree to it, but not all*’. Others asserted that advertising is ‘*prohibited in medicine*’, and that as a consequence, healers should not be using it either.

Several things are at stake in this ambivalent discourse around advertising and traditional healing. First, an example of the liveliness of law: the letter of the law itself is exceeded by daily practices and vernacular interpretations, that stretch it to new territories even where those are not strictly captured by the original scope of the law (Hertogh, 2004). Second, the choreographies are shaped by the ambivalence of traditional healing, between claims of its identity as ‘medical’, to the extent that it is expected to submit to some of the rules applied to biomedicine, and as Other. Finally, the restrictions on advertising placed upon medicine also echo professional norms that are shared among healers, in particular their own wariness towards those who seek to profit from their healing practice. Here, it is harder to distinguish to what extent these rules may be influenced by the law or by biomedical practice, and to what extent they stem from other customary norms that happen to share some elements with state law (Moore, 1973; Merry, 1988). State law, professional norms, and customary expectations overlap, shaped simultaneously by internal and external dynamics, and operate both independently from, and in conversation with, biomedical professions and their own expectations.

Overall, the current legal framing of traditional healing is an uneasy settlement. The ambivalence that permeates it, coupled with the lack of formal recognition by the state of a legal space for healers, creates a degree of precarity, while also preempting healers from acting against those they see as breaching professional norms. A notable feature of the current system, on which these patterns of fragility rest, is the limited input from the state. Boundary-drawing is left mostly to professionals, and in practice falls largely upon the Ordre des Médecins. Yet, traditional healing is more than a matter of health practice that can easily be delegated to doctors as experts. Instead, its ordering calls for a broader range of perspectives and expertise, reflecting its ontological and socio-cultural ramifications.

## Reopening the Fragile Settlement: Towards Formal Regulation?

In 2017, the government put forward a legislative proposal to revisit the ordering of traditional healing^4^. In doing so, it acknowledged the fragility of the current system, and the need for state intervention in this area of socio-political significance. Though adopted by the Council of Ministers, the draft was never debated in Parliament, triggering instead intense public debates between biomedical associations, healers and policy-makers. To this date, its formal proceedings have not restarted, stalled by the intensity of the controversy, and later the impact of the COVID-19 pandemic on state institutions. It remains seen, however, as a question that needs to and will be reopened, whether in its current form or an alternative. For the purpose of this paper, the controversy itself is as relevant as its outcome: it exposes further the epistemological and institutional tensions that lie at the core of the regulation of healing. Before turning to the significance of this controversy, and what it suggests of the fragile settlement that characterises the current, informally regulated, field of healing, we briefly contextualise the proposed legislation.

The drive behind the legislative proposal built onto various imaginaries of what traditional healing is about and could be about, adding a layer of complexity and contradictions to everyday practice (see in another context, Wahlberg, 2006). It also responded to demands from healer communities and institutions. Despite their differences, healers and their associations tend to find common ground in the desire to triage traditional healing practice, disentangling ‘genuine, knowledgeable’ healers from ‘quacks’. In that respect, the legislative proposal was seen by all healers we interviewed as a useful step towards improving their field of practice, and media coverage also suggests this is a widely held position. As one healer explained:

‘*When we were told that there was this new legislative draft, we thought we should get behind it because at some point when someone knows what they are doing, and are confident, they shouldn’t hide anymore. We decided to go and engage, and purify the field. We fought to clean the field, and we asked for the law to bring to justice those who are acting foolishly. That way, we would get rid of those who don’t know what they are doing, and let those who know support the population. Put an end to charlatanism*.’

Public health agents echoed this vision of the law as a tool to organise the field along lines healers were keen to draw: *‘We need to support them by passing this law, so we can distinguish those who do good work and help them. The others, we need to get rid of them because they ruin the system’* (public health official). Doctors, as we return to, read the proposed intervention of the state very differently. Before turning to their reaction, we briefly summarise the substance of the proposed text.

Adopted in the Ministerial Council in 2017, the Bill was considered even by its supporters as a starting point, sketching some general principles rather than the procedural details that would determine its practical workings:

‘It is only the start. This law is not going to solve everything. It gives a general framework, but we will need to adopt follow-up texts, much more targeted, for the next steps. I mentioned the issue of training. We don’t know where healers train, what they do. We need to figure it out. The law is the beginning, we need a foundation to build the rest but it won’t solve everything. In fact, if you use it initially to give penalties, you will be dishing out penalties to everyone because nobody is clean.’ (Public health official)

The Bill undoubtedly left numerous questions open, triggering, for some, a degree of scepticism or concern. At the same time, the principles it embedded already drew certain paths over others, suggesting shifts from current practice, as well as some continuity. For example, an immediate and most symbolic effect of the proposed law would be to explicitly legalise the practice of traditional healing (defined through a typology, ranging from herbalists to - more controversially - ritualists), taking out existing ambiguity about if and when it can be constitutive in and of itself of illegal medicine. Its legality would be subject to specific conditions of practice, some of which echo the lines of professional legitimacy drawn by healers themselves. For example, each healer would be authorised to practice in specific domains (eg. herbal medicine) or for particular types of pathologies, thereby limiting the type of claims that healers could make – including the more wide-ranging and controversial type. At the same time, such categorisations may not easily map onto the complex layers of healing practice, and leaves open the question of who would do this mapping.

Rather that entirely breaking away from previous practice, the proposed legislation would entrench some of the boundaries that currently constitute sites of friction between healers and the Ordre des Médecins: advertising by healers would be prohibited, and healers would also be forbidden from writing medical prescriptions. To this extent, its substance seems broadly aligned with current practice, and in fact clarifies some of the ambiguity that creates frictions between healers and doctors.

But breaking with continuity, the monitoring of these new rules, and of traditional healing as a whole, would be subject to a new institutional apparatus: the text proposed the creation of a *Conseil National des Praticiens de la Médecine Traditionnelle* (National Council of Traditional Healers), that would oversee the application of the law and participate in articulating relevant procedures and criteria further. This would effectively shift away the everyday monitoring from the Ordre des Médecins and towards a selected group of healers, rewriting institutional dynamics and their epistemological foundations. Maybe unsurprisingly, this would become a particular point of tension with the Ordre.

Shortly after its approval by the Ministerial Council, and before it was even formally tabled for a vote in Parliament, the proposed legislation was vehemently and publicly decried by the Ordre des Médecins and other associations of health professionals, in a carefully organised press conference (eg. Senenews, 2017). The proposed legislation had turned into a site of controversy, bringing to a head longer-standing debates about the place that traditional healing should occupy in contemporary Senegal. The objections expressed were layered, including substantive concerns with the formal legitimation of traditional healers; procedural frustration with the lack of involvement of the Ordre in the drafting process; and institutional concerns about the creation of a new Council, effectively replacing their own role as arbiter of the boundaries of legitimate healthcare. These objections also overlapped, and suggest more fundamental tensions around the interface between law and scientific knowledge in healthcare, and questions of epistemological control over the provision of care and their institutional implications. For one representative of the Interordre des Professionnels de Santé (an instance representing jointly doctors, pharmacists, dentists and vets):

‘This is the problem of the creation of a council of traditional healers. The Interordre reacted to this to oppose it, and to ask that all actors be involved, not just traditional healers. And by all actors i mean doctors, pharmacists, dentists, users, everyone. [The government] will need to justify why they want to create this institution.’

This concern was expressed alongside a frustration that the biomedical professions had not been consulted in the drafting process:

‘we were going to find out on TV that there was a legislative draft on traditional healing! […] In principle this draft should have gone through us, or at least the Ministry should ask for our opinion on this, because it is hugely significant to adopt rules that are about a medical practice, without going through the Ordre des Médecins. For us this is very serious. No country in the world with any self-respect would do this. We cannot adopt a law on medical practice without the opinion of the highest medical institution in the country. […] We are the highest medical authority in the country. People get confused: the Ministry is the highest political authority, in relation to health, but the highest professional authority is the Ordre des Médecins, but unfortunately people still don’t get it. I think that public authorities do not get what the role of the Ordre is, and maybe that’s why the President does not think it is necessary to speak to us.’ (Representative of the Ordre des Médecins)

Two interrelated elements are at stake in these statements. First, a deep resistance to the idea that traditional healers could be considered a professional and epistemic authority, regulated through rules laid out by the state and enforced by an institutional body of peers (reflecting, for example, the self-regulation of healthcare professions). Second, a deep-rooted attachment to the role of the Ordre des Médecins as a political actor in the field of public health, and a certain anxiety about the possible erosion of its institutional influence. Both echo the broader ambivalence around where traditional healing should sit vis-à-vis biomedicine that already plays out in the absence of formal regulation, but also suggest doctors’ resistance to it being positioned as anything other than subaltern to biomedicine.

If one key effect of the law, for its proponents, would be to ‘tidy up’, order and regulate the field of traditional healing, others are concerned that it would represent a formal endorsement by the state of practices that they see as either inherently problematic, or as needing external oversight and a reframing along scientific lines. For those concerned, if traditional healing is to be legitimated through law, it should also be purified, reorganised and overseen by biomedical knowledge and agents:

‘If you look at the definition they give, and that not everything can be proven, by intuition or whatever, in really blurry ways, anyone can be a traditional healer. It is opening the door to anything. […] We can’t be against traditional healing because phytotherapy is like the mother of medicine. But I think we could organise them, organise a training in phytotherapy, with pharmacognosie, botanics. So that people who are interested in traditional healing go to university and learn about it’ (Public health official)

‘It is a hugely valuable resource, that could guarantee the therapeutic sovereignty of our population, but it has to be under the control of scientists. Professionals, under the oversight of scientists.’ (Representative of the Ordre des Pharmaciens)

Yet, such conditioning of traditional healing to its folding within biomedical knowledge and institution is not without political implications: it suggests an assumption of hierarchical positioning of ontological orders, with the universality of science trumping other ways of knowing or being (Cant, 2020). In Senegal, such tension solidified around the question of authority over practice. By creating a new Council for traditional healing, the law would effectively also redefine the jurisdiction of the Ordre des Médecins, and the relationship between biomedicine and healing as fields of knowledge. The question of formal recognition by the state, and of the conditions of such recognition, primarily played out around the matter of jurisdiction, rather than substance. In fact, in its substance, the new law would have embedded the key principles that the Ordre uses everyday to determine where the boundaries of (illegal) medicine have been crossed. The response from the Ordre to the proposal suggests that institutional oversight is the more sensitive matter: taking away the authority of the Ordre as the de facto source of oversight over traditional healers would create a deeper shift in control over knowledge, possibly leading to a longer term rewriting of the respective positioning of biomedicine and traditional healing in the collective imaginary.

Tellingly, around the time that the draft legislation was submitted to Parliament, a group of doctors proposed their own alternative text (devoid of any formal legal significance), in which this alternative vision whereby traditional healing would remain institutionally dependent on biomedicine, is fully articulated^5^. It undoubtedly presents some similarity with the Ministry’s legislative draft, in particular in setting out specific criteria for the legality of practice of traditional healing. Yet, the institutional set up it proposes is radically different, illustrating further where the substantive concerns seem to lie: in this alternative proposal, the Ordre des Médecins would remain in charge of overseeing traditional healers, according to its own code of professional medical practice, rather than these powers being allocated to a Council of Traditional Healers.

Unsurprisingly, these demands of doctors, explicit in this letter but also expressed, sometimes more implicitly, in broader discourses, caused frustration among healers:

‘They want to be modern doctors and control traditional healing! We are going to fight this. We will never accept it. They are doing everything they can, but we will not accept it. We need to take care of our medicine. They can look after theirs, and we will look after our own! […] We need to elect people who work in traditional medicine to oversee our practice. […] Each to their own domain. They should stick to their own domain and support us so that we, also, can practice within our domain and modernise it.’

Overall, in these institutional frictions, two visions of traditional medicine are proposed that are not fully reconcilable: on the one hand, traditional healing is being sketched by the proposed legislation as a field that could potentially be regulated in its own terms, through an independent process of professionalisation and that would rely on the oversight of selected peers. Agents and institutions of biomedicine are not particularly relevant to this vision: the sphere of practice that they occupy would coexist with that of traditional healers, yet be considered for regulatory and professional purposes as being distinct (Akerele, 1987). This vision is broadly supported by healers, although the difficult question of how, and by whom, representative peers would be selected has not yet been addressed, and is likely to be complex given the diverse nature of traditional healing, and its internal tensions (see also Street, 2016). On the other hand, medical doctors and other biomedical professionals envisage traditional healing as a subaltern form of quasi-medical practice, that lacks the credential to elevate it as an independent sphere of healthcare practice, yet shares sufficient jurisdictional grounds with biomedicine for it to remain under its institutional purview. Underlying this position is a rejection of the ontological claims that traditional healers put forward as an alternative to the registers of science and medicine, as traditional healing’s own identity balances precariously between tradition and modernity.

A feature of the 2017 controversy is that the positioning and maintenance of traditional healing vis-à-vis biomedicine is entangled with legal and institutional inheritance, illustrating the difficulties for states to trigger legal change in areas where everyday legality layers formal and informal norms, each with longstanding and multiple roots (Merry, 1988). With current practice shaped primarily, as far as law is concerned, by the boundaries of illegal medical practice, as monitored and interpreted by the Ordre des Médecins, the possible futures of traditional healing are constrained by long-standing legal scripts and their institutional ramifications. The jurisdictional change proposed in 2017 would create a new institutional configuration, in which both the law on illegal practice, and the Ordre des Médecins, would see their influence over traditional healing shared with new actors, and somehow dissolved. This would foster a different kind of medical pluralism and indeed legal pluralism from that currently installed. Ultimately, the impossibility to reconcile different visions of who should have legal authority over the boundaries of healing was arguably the most significant factor behind the halting of the legislative process. Until such tension is resolved, traditional healing remains caught in a double-bind, expected to comply with a quasi-medical identity in some respect, while differentiating itself clearly from medicine in others, in order to remain within the borders of a plurality of rules.

## Conclusion

The fragile regulation of traditional healing in Senegal illustrates both the ongoing pressures to legislate in this field, and the difficulty of doing so. Even if new regulations are adopted, it is unclear that they would have significant effects in practice: regulating such diffuse practice as traditional healing, which by nature takes place in a multitude of spaces in which the state has limited access, is difficult to implement. In addition, even if adopted the text of the 2017 proposal leaves many difficult questions unanswered. For example, even if remaining focused on institutional matters, the divided landscape of healing in Senegal suggests that issues of membership and representation in a traditional medicine council would be complex. However, the stakes of the law are also located in the symbolic positioning of traditional healing in the Senegalese landscape, and arguably of the symbolic socio-political positioning of Senegal as a contemporary state. Those who are most invested in favour of or against the law are also seeking to make claims that go beyond the everyday practice of traditional healing (and arguably have little to do with it). Here debates hinge on the interface between ‘regulation’ and ‘recognition’ that is at the core of similar debates beyond Senegal, and familiar to other states that have engaged in such endeavours: while the law is at one level about defining the conditions for the legal practice of healing (its regulation), it is also a placeholder for broader tensions about its recognition. This means at least two things: the biomedical professions see such recognition as an encroachment on their practice, and potentially a threat to the scientific consensus they view as essential to modernity. And the significance of the law is maybe highest for those who are most invested in the symbolic value of recognition for traditional healing, and identities.

While it is unclear whether and in what form legal regulation of traditional healing as proposed in 2017 will take shape, the matter is likely to remain complicated by the ambivalent positions that healers occupy in the healthcare landscape, and their fragile relationships with doctors. In their day-to-day experience, healers are seen by doctors as being both ‘fakes’, illegally practicing an impoverished form of medicine, and belonging to a different sphere of practice. They are considered Others, to be triaged and pushed aside as quacks and charlatans, yet sufficiently relevant to biomedical practice to be maintained under its oversight. The jurisdictional position of traditional healing is complicated by this uncertain position between genuine Other and fake look-alike, imitating yet displacing notions of care and healing. Under the current system, if healers are subject to repeated calls to be scienticised and modernised, their own attempts at using the tools of diagnosis of biomedicine also exposes them more acutely than practices that can be neatly seen as traditional. Using X-rays, blood-tests, and the other diagnostic tools of biomedicine is read as a more obvious attempt to encroach into the territories of biomedicine. Rather than this being purely a matter of medical pluralism, and its sometimes uneasy shapes, this difficult coexistence is also directly linked to the long-standing legal ordering of the field. Caught between laws designed for biomedicine only, its fluid interpretation by both state and non-state actors, and its entanglement with other forms of professional and customary norms, traditional healing has come to be defined through its coexistence with biomedicine, in law and in practice. Any attempt to legislate will need to reopen the discreet institutional and normative arrangements in which actors on all sides have embraced a certain ambivalence, finding some common ground in a desire to order and tidy up the field of practice, yet disagreeing on the symbolic and political directions that any new ordering should take.

## Notes

1. To contextualise such framing, see Cloatre and Ashworth, 2022.

2. Loi n° 2017-27 du 13 juillet 2017 portant Code de la Presse.

3. Lettre du Président de l’ONMS au Président du CNRA, du 15/04/2019.

4. Projet de loi relatif à l’exercice de la Médecine Traditionnelle au Sénégal, adopté en Conseil des Ministres le 31 Mai 2017.

5. Unpublished letter, provided by the Ordre des Médecins during one of our interviews.
